# A Tree-Like Model for Brain Growth and Structure

**DOI:** 10.1155/2013/241612

**Published:** 2013-09-02

**Authors:** Benjamin C. Yan, Johnson F. Yan

**Affiliations:** ^1^Yan Research, P.O. Box 4115, Federal Way, WA 98063, USA; ^2^University of Illinois College of Medicine, 190 Medical Sciences Building, MC-714, 506 Mathews Avenue, Urbana, IL 61801, USA

## Abstract

The Flory-Stockmayer theory for the polycondensation of branched polymers, modified for finite systems beyond the gel point, is applied to the connection (synapses) of neurons, which can be considered highly branched “monomeric” units. Initially, the process is a linear growth and tree-like branching between dendrites and axons of nonself-neurons. After the gel point and at the maximum “tree” size, the tree-like model prescribes, on average, one pair of twin synapses per neuron. About 13% of neurons, “unconnected” to the maximum tree, migrate to the surface to form cortical layers. The number of synapses in each neuron may reach 10000, indicating a tremendous amount of flexible, redundant, and neuroplastic loop-forming linkages which can be preserved or pruned by experience and learning.

## 1. Introduction 

 The molecular weight distribution (MWD) of polycondensation of branched-chain monomers of the type RA_*f*_ has been derived classically by Flory [1] and generalized by Stockmayer [2]. Here, *f* is the number of functional groups, or “functionality” of group A. Mathematically, this widely quoted distribution function has been treated as power-series distribution and compound distribution that provides a simple concept; that is, single-parameter expressions of number- and weight-average “degrees of polymerization” (DP) are sufficient to generate the entire MWD for branched polymers [3]. Moreover, using a cascade formulation involving functionals and probability generating functions (PGF), this distribution can be extended to finite systems [4].

Here, the previously derived properties of this finite distribution are applied to synapse formation in the brain. A neuron has multiple dendritic processes and an axon, which can also be branched. Neurons are generally three or more orders of magnitude greater in size than molecular units. However, the functionality of a neuron may be 10^3^ times larger than that of typical branched molecules (*f* = 10000). This large functionality also means there is great accessibility to the connection sites, and the long flexible axons offer a favorable condition for connection between neurons. The “tree-like,” or “ring-free,” assumption in the Flory-Stockmayer theory can be satisfied by the initial linkage of head-to-tail linear chains and followed by a “tree-like branching.” A neuron itself can be considered a small tree. Similarly, the peripheral nervous system (PNS) also resembles a tree made of the nerve bundles, which can be as large as 1.5 meters. The Finite Flory-Stockmayer theory (FFST) deals with numbers of highly branched repeat units and their association-dissociation mechanism. Therefore it is applicable to the statistical treatment of brain growth, neuronal connectivity, and information transmission. Linear growth and subsequent tree-like branching allow two simple equations to be applied up to a maximum tree size constrained by the FFST.

In practical, real and natural systems, lignin in wood is a tree-like molecule [5], and a major class of corals is also tree-like [6]. Thus the applicability of the F-S theory in molecular, cellular or animal size scales is equally sound if the repeating branched units can be properly identified. 

 Early brain growth is very robust and rapid. In the human brain the crosslinking between neurons and the growth of the linked neurons (polyneurons) proceed with a rapid rate. Because of their highly branched dendrites and axons, there are three types of crosslinking: axodendritic (A-D), axosomatic (A-S), and axoaxonic (A-A) synapses, in the order of decreasing abundance. In addition, there are at least three forms of crosslinks. The first are end-links which are links between the tips of an axon and a dendritic spine. The second form is a split-end link, which is shaped like the Greek letter *Ω* on top of *V* (the shape of a pair of dendritic spines), designated *Ω*/*V*-linking. The latter conformation represents a pair of twin synapses. The third form is the most abundant “*X*” shaped, mostly A-D linkages.

 Structural and functional features of neurons set the limits and modes of various crosslinks. For example, there is no dendrite to dendrite (D-D) synapse because dendrites have been shown to demonstrate self-avoidance even in fruit fly brains [7]. With respect to a growing polyneuron tree, the “ring-free” assumption is also a self-avoidance rule. A conceptual illustration furnishes a visual model of a pair of *Ω*/*V*-twin synapses by removing the axonic “spaghetti maze” for a clearer view [8]. 

 The rate of growth and rate of crosslinking in the brain occur exponentially in the months before and after birth in the human brain [9] and in the cortex of laboratory animals [10]. Initial linear growth and tree-like branching appear to be the most efficient path-way to grow and expand rapidly in a confined or open space [11]. 

 The following is an ideal human brain model with neurons assumed to be homogeneous, with identical size, shape, and number of crosslinking sites. 

 There are two basic assumptions in the FFST. First, all linkages (synapses) are formed with equal probability, or the same “extent of reaction.” Second, no ring is formed between branches of a growing chain. The second assumption is also termed the “tree-like model” [4]. The brain growth process is discussed with limiting forms of equations derived previously, and upper bounds of the variables and parameters are strictly constrained. 

## 2. Limiting Equations and Numerical Examples

Symbols and abbreviations derived previously [4].

 
*N* = system size = number of neurons (in a brain or a “module”).
 
*f* = functionality = number of connection sites per monomer (neuron) = 10000 or in thousands [11].
 
*k* = number of generations in the “transformer” probability generating functions (PGF) in the cascade formulation [4].
 DP = degree of polymerization = number of connected neurons in a growing chain = *x* = 1,2, 3,…, *N*.
 
*α* = extent of reaction or fraction of connected synapse sites.
 
*β* = (*f* − 1)*α* which is approximately *fα* for large *f* = crosslinking index.
 〈*x*_*n*_〉 = number-average DP.
 〈*x*_*w*_〉 = weight-average DP.
 
*β*_*c*_ = 1 is value of *β* at gel point.
 
*β*_*m*_ = 2, maximum value of *β* allowed in tree-like model.
 
*g*_*m*_ = gel fraction at *β*_*m*_.

At large *f* values, most of the finite system equations derived previously [4] become independent of *f*. The FFST is represented by the two averages:

(1a) 〈xn〉=(1−fα2)−1

(1b) =(1−β2)−1 for  large  f,

(2a) $$\begin{array}{c} \langle x_{w}\rangle \left(\beta \right)=1+f\alpha \left(1+\beta +\beta^{2}+\cdots +\beta^{k}\right). \end{array}$$

The last equation is derived and proven in Supplementary Material session available online at http://dx.doi.org/10.1155/2013/241612. In its application, (2a) can be written in a more compact form:

(2b) $$\begin{array}{c} \langle x_{w}\rangle \left(\beta \right)=1+\frac{\beta \left(1-\beta^{k+1}\right)}{1-\beta },\;\beta >1\;\text{or}\;\beta <1. \end{array}$$

In particular,

(3) $$\begin{array}{c} \langle x_{w}\rangle \left(\beta_{c}\right)=1+\left(k+1\right),\;\beta_{c}=1. \end{array}$$

The maximum value for 〈*x*_*w*_〉 is *N*, that is, a giant gel particle with *N* neurons all connected in this “polyneuron” tree. With this value for 〈*x*_*w*_〉 at *β*_*m*_ = 2 in (2b), the result is simply

(4a) $$\begin{array}{c} N=2^{k+2}. \end{array}$$

Equation (4a) expresses the system (brain) size in terms of the number of cell division, as powers of 2. The maximum value of 〈*x*_*n*_〉 is also *N*, when using a precise value [4] of *β*_*m*_ = 2(1 − *N* ^−1^). 

 The unconnected monomer neurons have a weight fraction of *w*(1) which can be approximated with the weight fraction in an infinite system [4] as

(5) $$\begin{array}{c} w\left(1\right)={\left(1-\alpha \right)}^{f}=e^{-\beta }\;\text{for}\;\text{large}\;f. \end{array}$$

Equation (5) provides a physically measurable definition for *β*. Since this monomer fraction does not join the tree growth scheme, the maximum “tree-like” value for *β* is 2 and the attainable DP for the gel particle is (1 − *e* ^−2^)*N*, which may be applied to the left-hand side of (4a)

(6) $$\begin{array}{c} \left(1-e^{-2}\right)N=2^{k+2}, \end{array}$$

which serves as a correction for computing *k*. But this is only a minor correction; for example, for *N* = 10^11^, *e* ^−2^ = 0.135, (4a) gives *k* = 34.54 and (6) yields *k* = 34.33 (Cf.  Table 1). The gel fraction 1 − *e* ^−2^ = 0.865 is denoted as *g*_*m*_. 

The case of *N* = 10^11^ and *f* = 10000 in Table 1 is the high-functionality system of the human brain [11]. The pregel distribution or the distribution in the sol fraction in Table 1 is narrow, with a dispersion ratio or dispersity *r* (ratio of weight-average DP to number-average DP) very close to 1, even for a system of 10^11^ neurons. Equation (5) indicates that the sol fraction contains mainly unconnected monomer neurons. These free, unconnected mononeurons may form other classes of aggregate structures, such as cortex layers (horizontal sheets) and minicolumns (vertical sheets) [11], or they may join the gel in a nontree fashion, as *β* reaches hundreds or even thousands. 

## 3. Postgel Relations with Extensive Ring Formation

 As *β* > 2, ring formation in the gel particle cannot be avoided [1]. Indeed when *β* = ln⁡(10^11^) = 25, only 1 in 10^11^ neurons remains in the sol fraction. In a human neuron with *f* = 10^4^ available connection sites, *β* may reach a large value of 8000. In this case, there is a near-zero chance of finding a free neuron that remains unconnected. The large values of *β* are a measure of excessive numbers of rings or loops. Information transfer is efficient when it takes a tree-like path in such a sea of ring structures. 

 This theory thus provides a statistical, nongeometrical model for brain growth, neuron packing, and neuron firing. Geometrical description such as calculation with cell and branch volumes tends to ignore the fact that in chemical solutions or cellular suspensions, volumes of cellular components are not additive [11].

 In the postgel range of 1 < *β* < 2, tree-like growth reaches a maximum size determined by (6). At *β* > 2, monomer neuron fraction can be considered as the sol fraction *s*. Thus the approximation

(7) $$\begin{array}{c} s=w\left(1\right)=e^{-\beta } \end{array}$$

or the gel fraction

$$\begin{array}{cc} \left(7^{*}\right) & g=1-e^{-\beta } \end{array}$$

is a good approximation for large *β*. Indeed the plot of *g* versus *β*, in the entire range of 0 < *β* < *f*, is remarkably similar to a “brain growth” curve [12]. This kind of “growth curves” can also be depicted as developmental curves of spine densities in the cortex of rat, mouse, and guinea-pig [13]. 

 The fractions of free and unconnected neurons, *w*(1), being *e* ^−1^( = 0.368), and *e* ^−2^( = 0.135) at *β* = 1 and 2, respectively, are in exact agreement with those obtained by using a simple binomial distribution for the probability of connecting neighboring cortical neurons at the binomial averages [10] of 1 and 2. This agreement is not surprising since the PGFs of the originating “root” (zero generation) and subsequent generations of *k*-fold compounding all take the binomial form (Supplementary Material). 

The asterisk on ((7*)) denotes that it is a relation that holds well beyond gelation in the presence of excessive rings. In fact, denoting the sol and gel properties by and after the gel point, the size distribution becomes heterogeneous and splits into two very narrow peaks at *w*(1) and around *N*, with [4]

$$\begin{array}{cc} \left(8^{*}\right) & \frac{1}{\langle x_{n}\rangle }=\frac{s}{\langle x_{n}^{\prime }\rangle }+\frac{g}{\langle x_{n}^{\prime \prime }\rangle }, \end{array}$$

$$\begin{array}{cc} \left(9^{*}\right) & \langle x_{w}\rangle =s\langle x_{w}^{\prime }\rangle +g\langle x_{w}^{\prime \prime }\rangle , \end{array}$$

$$\begin{array}{cc} \left(10^{*}\right) & \beta =s\beta^{\prime }+g\beta^{\prime \prime }. \end{array}$$

The FFST defines an *x*-mer as having (*x* − 1) linkages without any loop structure. The maximum tree size has a DP of *N* neurons with *N*, or precisely (*N* − 1), linkages. At this maximum size, *β* = 2, and (2a) reduces to (4a) as a constraint set by the system size. In the entire range 0 < *β* < *f*; however, *β* is also the number of linkages per neuron. 

If *N* pairs of twin synapses are uniformly distributed along a linear *N-*meric chain, it would then serve as a backbone for the entire system of connected neurons. This scheme leaves all the loop-forming *X*-linked connections in the branched, nonlinear chains. However, uniform distribution appears unlikely because at *β* = 2, there is still a fraction of *e* ^−2^ or 13.5% (by weight) of neurons remained unconnected at the maximum tree size. This is approximately the same fraction as that of human cortex [14]. 

 In a given neuron, there may be 10000 synapses that are formed by 10000 incoming post-synapses and 10000 outgoing presynapses [11]. The total number of linkages is *Nβ*, and the total redundancy is defined as

(11) $$\begin{array}{c} D=N\left(\beta -1\right)\;\text{for}\;\beta >2. \end{array}$$

For *β* = 2, there is a redundancy of *D* = *N* linkages. Of these 2*N* linkages at the maximum tree size, there are *N* tree-like linkages paired by exactly *N* loop-forming linkages. This seems contradictory, because in a strict sense of the ring-free assumption in FFST, a *Ω*/*V*-linkage, or a pair of split-end linkages, is a smallest loop. Such a pair of twin synapses stabilizes a crosslink or the entire network. For *β* = 8000, almost all 8000*N* redundant linkages provide not only greater redundant security, but also greater neuroplasticity to the gel-like network structure of the brain. At the early explosive growth stage of synaptogenesis [9], end-linking or specially the *Ω*/*V*-linking may be a preferred mode for linear and tree-like branching chain growth (Table 1). In this sense, each tree-like linkage is considered as having a pair of twin synapses. 

 Redundant, loop-forming linkages do not contribute to the DP of a growing chain. Thus the maximum-sized gel remains at a DP of *g*_*m*_ *N*, instead of the *N* discussed above. The maximum number-average DP is also *g*_*m*_ *N*, rendering the dispersity at 1. Finally the unconnected or cortical fraction of neurons should also join this growing gel, approaching a final DP of *N*, where the tree-like equations (1a), (1b), and (2a) no longer hold, but ((8*))–((10*)) are still valid as *β* → *β*′′ → *f*. This is a state in which neurons in sol-sol, sol-gel, and gel-gel are all linked. This is the complete gelation scheme proposed by Flory [1, 4], which differs from that of Stockmayer and others [4].

## 4. Neuron Wiring in the Brain

The simplest strategy for neuron wiring is a snake-like strategy as depicted in Figure 1(a) of [11], in which a long wire (axon fiber) is connected to all 209 neurons in that figure. In a “10000 nearest neighbors” model, the long wire makes a single connection to each of the 10000 light bulbs tangentially, meaning only 1 point of contact at each bulb.

Since a snake does not bite its own tail, the snaking scheme is ring-free.

 The wiring model proposed here for the connection of *non-cortical* neurons may be called concentric “hardball” and “softball” model, obtained by invoking the above tree-like definitions, numbers, and equations, plus 3 types and 3 forms of synapses, and at least 2 self-avoidance rules.

### 4.1. The Hardball Is Where Most of the Neuron Mass Concentrated

It is centered at the soma of a given neuron, with a radius *R*_1_ encompassing the entire soma, plus axon and dendrite branches near the soma. All 3 cell components (S, A, and D) can provide postsynapses to an incoming presynaptic axon. All 3 types (A-S, A-A, and A-D) of synapses may be present inside or on the surface of this hardball. The hardball is a (relatively) solid, rigid, and stationary target for the incoming straight-shooting pre-synaptic axon tip [10]. 

Incoming axons can be envisioned as straight arrows, each with a long string attached to its end [10]. In the early stage of synaptogenesis, an axon arrow is shot toward the hardball target, most likely with 0 or 1 hit [10].

 The random hits to the hardball can be described with a binomial distribution:

(12) $$\begin{array}{c} b\left(y;np\right)=\left(\frac{n!}{y!\left(n-y\right)!}\right)p^{y}{\left(1-p\right)}^{n-y},\;y=0,1,2,\ldots , \end{array}$$

where *b*(*y*; *np*) is a binomial distribution function, with random variable *y* and parameter *p*. But *n*, the available target sites, is a large constant. The PGF and the mean are

(13) $$\begin{array}{c} \phi \left(\theta \right)={\left(1-p+p\theta \right)}^{n},\\ \phi^{\prime }\left(1\right)=np. \end{array}$$

Note that the random variable *y* starts with 0, and *θ* is the dummy variable [4] of the PGF. At low *np* values, for example, *np* = 1, the distribution is broad, with twin peaks at *y* = 0 and *y* = 1. This means the most probable outcome is 0 or only 1 hit [10]. The cases for *np* = 1 and 2, with *b*(0; *p*) = *e* ^−1^ and *e* ^−2^, have been discussed above as the fractions of “unconnected neurons” at these averages.

The simple binomial PGF in (13) and its distribution are different from FFST size distribution in other aspects, besides the similarity in fractions *e* ^−1^ and *e* ^−2^. The simple binomial distribution is for the random hits because it has nothing to do with size of aggregates or polyneurons since there is no gelation. This “zero-or-one” scheme, proposed for random hits on linking of mouse cortical neurons [10], actually fits the principle of cascade formulation on tree-like connection of non-cortical neurons [4], which is also employed in the present treatment of ring-free assumption up to the limit of maximum tree size. 

 A more recent estimate is a narrow peak at *np* = 5 for linking two neighboring cortical neurons [14]. This means the distribution is peaked around *y* = 5, but the *y* = 0 peak is no longer significant. That is, with an expected 5 linkages between two cortical neurons, there is practically no chance for a “no hit.” These probabilistic arguments suggest that the connection of cortical neurons is much more orderly or structured than that in the initial bulk phase of tree-like connection.

 Contrary to the simple binomial probability distribution, the branched-chain size distributions in Table 1, for *f* = 3 or more, are narrow at low *β*, broadening with increasing *β* values and reaching a maximum at gel point [4] when *β* = 1. 

 It can be further simplified by assuming that synapses formed onto the hardball are entirely of the twin synapses of the *Ω*/*V* form, for the following reasons. Firstly, the rough, spiny, and more abundant dendrite branches provide conditions required for twin synapse formation. Secondly, even with a smooth and less active surface on the soma, the A-S connections may stabilize themselves with such twin linkages. All *Ω*/*V*-linkages in the hardball are designed to provide a “skeleton” for a brain lasting for a life-time of the host animal [9]. 

### 4.2. The Softball Layer

In addition to the long, thin axons, the thick layer outside the hardball, of radius *R*_2_ from the central soma, is mainly composed of flexible and distal portions of dendrite branches. Incoming axons shot through this flexible, ribbon-like cloud have nonstationary targets for contact. Connections to this softball region are made with nontree, redundant *X*-links. A given neuron has up to 10000 contact sites for 10000 axonic presynapses from other neurons. If the synaptic density (in numbers of synapses per unit volume) is uniform in both hardball and softball regions, then

(14) $$\begin{array}{c} \frac{\left(R_{2}^{3}-R_{1}^{3}\right)}{R_{1}^{3}}=\frac{\left(f-2\right)}{2}, \end{array}$$

where *f* = 10000. And 2 is the number of synapses, of the *Ω*/*V*-form, connected to the smaller hardball. The result is simply *R*_2_ > 17*R*_1_, which is easily achievable to satisfy this large number [11] of *f*. 

## 5. Conclusions

For comparison with other statistical models, the FFST provides tree-like parameters such as *k* (an abstract concept of *k*-fold compounding in PGF), and *k* + 2 (as weight-average DP and as number of generations in cell divisions) at various crosslinking stages. Crosslinking index is *β* ≪ 1 for pregel linear growth; *β* = 1 at the gel point, and *β* = 2 at the maximum tree size. When 2 < *β* < *f* extensive ring formation occurs, as the observed range of number of synapses per neuron. 

Human cortical neurons are more homogeneous, less dense in synapses, and more ordered in their connection than the well-studied much smaller mouse cortex [10, 11]. In the human cortex, these advantages translate into a greater neuroplasticity and much greater freedom in the information transmission process. Low synapse density in non-cortical regions means an early onset of tree-like branching, so that the massive, late-occurring *X*-links can have sufficient space for high neuroplasticity. In this sense, the tree-like model proposed here is behaving as a “supporting” system—much like that animal skeletons providing support, with muscles and organs filling in, for their host bodies. 

The medium for neurons is well dispersed with glia cells and other mixture components. The brain growth in terms of brain volume or synapse density follows a general curve shape of gel development in ((7*)). Another developmental *β* versus time curve, in months before and after birth, surges with a rapid rate to a maximum *f* at 8 postnatal months. Thereafter, it declines and levels off at *f*/2 and continues to a very mature age of 70 years [9]. These surge and decline do not affect the shape of a growth curve because the values of *β* are in thousands. 

 The statistical model for a maximum tree gel leads to a physical model wherein all neuron units are connected entirely by *Ω*/*V*-twin synapses in the hardball region, accounting for 3 types and 3 forms of observed synapses. In the outer softball region highly redundant, flexible, accessible, and neuroplasticity are the characters of the *X*-linked synapses. In a brain, twin synapses are present in a much smaller amount than the *X*-links, but the twins are obviously more visible and easily identified [8, 11, 13]. 

 Perhaps the simplest brain is that of *C. elegans* [15], in which a large sensory neuron has only one pair of “horse-shoe” shaped dendrite branches. Thus, the prediction of “one linkage, twin synapses” from FFST holds even for this primitive animal. In higher animals, the observation of twin synapses [8, 15] indicates that they are relics of tree-like structure. 

## Supplementary Material

The molecular weight distribution (MWD) of polycondensation of branched-chain monomers of the type RA_*f*_. Here, *f* is the number of functional groups, or “functionality” of group A. Mathematically, this widely quoted distribution function has been treated as power-series distribution and compound distribution that provides a simple concept; that is, single-parameter expressions of number- and weight-average “degrees of polymerization” (DP) are sufficient to generate the entire MWD for branched polymers. Moreover, using a cascade formulation involving functionals and probability generating functions (PGF), this distribution can be extended to finite systems.

## Figures and Tables

**Table 1:** Properties calculated from a tree-like model for branched “polyneurons” with low (*f* = 3) and high (*f* = 10^4^) functionalities. At a pregel stage, *β* = 0.1 and at the gel point, *β* = 1. For *f* = 3, the accurate relation *β* = (*f* − 1)*α* is used, as in (1a) and other equations in [4].

			*β* = 0.1			*β* = 1		
*f*	*N*	*k*	〈*x*_*n*_〉	〈*x*_*w*_〉	*r*	〈*x*_*n*_〉	〈*x*_*w*_〉	*r*
3	10^4^	25	1.111	1.167	1.05	4	40	10
10^4^	10^11^	34	1.053	1.111	1.05	2	36	18
