# Supplementary material for: A Tree-Like Model for Brain Growth and Structure
Source: J Biophys. 2013 Sep 2;2013:241612. doi: 10.1155/2013/241612 (PMC3775399; doi:10.1155/2013/241612)
Supplement: Supplementary file 1 — The molecular weight distribution (MWD) of polycondensation of branched-chain monomers of the type RAf. Here, f is the number of functional groups, or “functionality” of group A. Mathematically, this widely quoted distribution function has been treated as power-series distribution and compound distribution that provides a simple concept; that is, single-parameter expressions of number- and weight-average “degrees of polymerization” (DP) are sufficient to generate the entire MWD for branched polymers. Moreover, using a cascade formulation involving functionals and probability generating functions (PGF), this distribution can be extended to finite systems. [file 241612.f1.pdf]

Supplementary material

Cascade Theory. This theory starts with the "root" or zeroth generation of the molecular tree, with a PGF

$$F_0(\theta) = (1-\alpha+\alpha\theta)^f$$

Where θ is the dummy variable in the PGF. The first and later generations must have functional groups reduced by 1, their PGF are

$$F_1(\theta) = (1-\alpha+\alpha\theta)^{f-1}$$

In a graphical illustration, the connected generations are represented as layers around one connected chain. The weight distribution of an x-mer has a PGF given by

$$W(\theta) = \theta F_0(u_k) = (1-\alpha)\, u_k(\theta) + \alpha\, u_k^2(\theta)$$

Where u_k is called the transformer PGF up to the kth generations:

$$u_k\ (\theta) = \theta F_1\ (\ldots(\theta F_1\ (\theta F_1(\theta)))\ \ldots) \qquad \text{(S1)}$$

So that there are k pairs of parentheses. That also means $u_k\ (\theta)$ is the k-fold compounding PGF. All of the above $F_1(\theta)$, $u_k\ (\theta)$ and $W\ (\theta)$ are PGF. As such, for the PGF $F_1\ (\theta)$, it has the following propeties: (i) Normalization condition: $F_1\ (1) = 1$; and (ii) the average or the mean: $F_1'(1) = (f-1)\ \alpha = \beta$..

Similarily, $F_0'(1) = f\ \alpha$ and $W'(1) = \langle x_w \rangle$ which can be shown to be

$$\langle x_w \rangle = 1 + f\ \alpha(1+\beta+\beta^2+\ \ldots.+\beta^\kappa) \qquad \text{(S2)}$$

which is also eq. (2) in the text.

Proof of eq. (2).

(a) The simplest case is k = 1 in eq. (S1)

$$u_1(\theta) = \theta F_1(\theta)$$

$$u_1'(1) = F_1'(1) + F_1(1) = \beta + 1$$

(b) By the method of mathematical induction, assuming

$$u_{k-1}'(1) = 1 + \beta + \beta^2 + \ldots + \beta^{k-1}$$

is valid,

then

$$u_k'(1) = F_1(u_{k-1}(1)) + F_1'(u_{k-1}(1))\, u_{k-1}'(1)$$

$$= 1 + \beta + \beta^2 + \ldots + \beta^k$$

which leads to eq. (2) on substituting into the expression for W'(1). The above derivation and proof are new.
